# Draft Genome Assembly of *Klebsiella pneumoniae* Type Strain ATCC 13883

**DOI:** 10.1128/genomeA.00939-14

**Published:** 2014-09-25

**Authors:** H. E. Daligault, K. W. Davenport, T. D. Minogue, K. A. Bishop-Lilly, D. C. Bruce, P. S. Chain, S. R. Coyne, K. G. Frey, J. Jaissle, G. I. Koroleva, J. T. Ladner, C.-C. Lo, L. Meincke, A. C. Munk, G. F. Palacios, C. L. Redden, S. L. Johnson

**Affiliations:** a Los Alamos National Laboratory, Los Alamos, New Mexico, USA; b Diagnostic Systems Division (DSD), United States Army Medical Research Institute of Infectious Diseases (USAMRIID), Fort Detrick, Maryland, USA; c Naval Medical Research Center (NMRC)-Frederick, Fort Detrick, Maryland, USA; d Henry M. Jackson Foundation, Bethesda, Maryland, USA; e Center for Genome Sciences (CGS), USAMRIID, Fort Detrick, Maryland, USA

## Abstract

*Klebsiella pneumoniae* is a common cause of antibiotic-resistant bacterial infections in immunocompromised individuals. Here, we present the 5.54-Mb scaffolded assembly of the type strain *K. pneumoniae* type strain ATCC 13883, as deposited in GenBank under accession no. JOOW00000000.

## GENOME ANNOUNCEMENT

*Klebsiella pneumoniae* is an encapsulated Gram-negative bacillus often found in healthy human mucosa but can cause high-mortality bacterial pneumonia in those with weakened immune systems. In fact, the species may cause up to 8% of nosocomial infections in the United States (1). *K. pneumoniae* ATCC 13883 is commonly used in research and diagnostics. It is the type strain and has type 3 antigenic properties.

High-quality genomic DNA was extracted from a purified isolate using QIAgen Genome Tip-500 at USAMRIID-Diagnostic Systems Division (DSD). Specifically, a 100-mL bacterial culture was grown to stationary phase and nucleic acid extracted per manufacturer’s recommendations. Sequence data were generated using a combination of Illumina and 454 technologies (2, 3). Specifically the Illumina library consisted of 100-bp reads at 542-fold genome-coverage and the 454 library consisted of long-insert paired-end reads (18-fold genome coverage, 7,658 ± 1,915 bp insert). The two libraries were assembled together in Newbler (Roche) and the consensus sequences computationally shredded into 2-kbp overlapping fake reads (shreds). The raw reads were also assembled in Velvet and those consensus sequences computationally shredded into 1.5-kbp overlapping shreds (4). Draft data from all platforms were then assembled together with Allpaths and the consensus sequences computationally shredded into 10-kbp overlapping shreds (5). We then integrated the Newbler consensus shreds, Velvet consensus shreds, Allpaths consensus shreds, and a subset of the long-insert read-pairs using parallel Phrap (High Performance Software, LLC). Possible mis-assemblies were corrected and some gap closure accomplished with manual editing in Consed (6–8).

Automatic annotation of the *K. pneumoniae* ATCC 13883 genome assembly utilized an Ergatis based workflow at Los Alamos National Laboratory (LANL) with minor manual curation. The final annotated assembly is available in NCBI (accession no. JOOW00000000) and the raw data can be provided upon request. Preliminary review of the annotation indicates ca. 10 genes each involved in lactam resistance and toxin/antitoxin roles. An in-depth comparative analysis of this genome in relationship to other members of the species is currently under way and will be published in a subsequent report.

### Nucleotide sequence accession number.

The final genome assembly for *K. pneumoniae* ATCC 13883 has been deposited in GenBank as accession no. JOOW00000000.
